# Results-based financing as a strategic purchasing intervention: some progress but much further to go in Zimbabwe?

**DOI:** 10.1186/s12913-020-5037-6

**Published:** 2020-03-06

**Authors:** Sophie Witter, Yotamu Chirwa, Pamela Chandiwana, Shungu Munyati, Mildred Pepukai, Maria Paola Bertone, Steve Banda

**Affiliations:** 1 ReBUILD programme, Queen Margaret University, Edinburgh, EH21 6UU Scotland; 2 ReBUILD and Biomedical Research and Training Institute, P O Box CY, 1753 Harare, Zimbabwe; 3 Biomedical Research and Training Institute, 10 Seagrave Road, Avondale, Harare Zimbabwe; 4 Policy and Planning, Ministry of Health and Child Care, Harare, Zimbabwe

**Keywords:** Results-based financing, Strategic purchasing, Zimbabwe, Maternal and child health care, Performance-based financing

## Abstract

**Background:**

Results-Based Financing (RBF) has proliferated in the health sectors of low and middle income countries, especially those which are fragile or conflict-affected, and has been presented by some as a way of reforming and strengthening strategic purchasing. However, few if any studies have empirically and systematically examined how RBF impacts on health care purchasing. This article examines this question in the context of Zimbabwe’s national RBF programme.

**Methods:**

The article is based on a documentary review, including 60 documents from 2008 to 2018, and 40 key informant (KI) interviews conducted with international, national and district level stakeholders in early 2018 in Zimbabwe. Interviews and analysis of both datasets followed an existing framework for strategic purchasing, adapted to reflect changes over.

**Results:**

We find that some functions, such as assessing service infrastructure gaps, are unaffected by RBF, while others, such as mobilising resources, are partially affected, as RBF has focused on one package of care (maternal and child health services) within the wider essential health care, and has contributed important but marginal costs. Overall purchasing arrangements remain fragmented. Limited improvements have been made to community engagement. The clearest changes to purchasing arrangements relate to providers, at least in relation to the RBF services. Its achievements included enabling flexible resources to reach primary providers, funding supervision and emphasising the importance of reporting.

**Conclusions:**

Our analysis suggests that RBF in Zimbabwe, at least at this early stage, is mainly functioning as an additional source of funding and as a provider payment mechanism, focussed on the primary care level for MCH services. RBF in this case brought focus to specific outputs but remained one provider payment mechanism amongst many, with limited traction over the main service delivery inputs and programmes. Zimbabwe’s economic and political crisis provided an important entry point for RBF, but Zimbabwe did not present a ‘blank slate’ for RBF to reform: it was a functional health system pre-crisis, which enabled relatively swift scale-up of RBF but also meant that the potential for restructuring of institutional purchasing relationships was limited. This highlights the need for realistic and contextually tailored expectations of RBF.

## Background

Over the last decade, the model of RBF in which funds to health facilities are made conditional on agreed outputs or outcomes, often with quality adjustments (sometimes also called performance based financing or PBF) [1], has been increasingly implemented in fragile and conflict-affected states (FCAS). While research and evidence on RBF has grown since the first systematic review of its application in low and middle income countries [2], there remain some very significant gaps in our understanding of it, in particular in FCAS, where there is a need to understand the programmes’ proliferation and how they may operate differently in these contexts [3], but also to examine their integration and wider system effects. RBF has been presented by some authors as a health system intervention with the potential to drive a more strategic approach to purchasing [4]. Others have suggested that it is likely that RBF could spur strategic purchasing reform during the ‘expansion’ phase when it becomes part of the system [5]. However, there has been limited empirical study of RBF’s impact on strategic purchasing in low and middle income (and especially fragile) countries.

Strategic purchasing is a core function of health financing, as defined by the WHO [6]. However, it is a relatively novel concept and a single, clear definition of it is still missing in the literature. A recent WHO policy brief states that, “purchasing refers to the allocations of pooled funds to healthcare providers for the delivery of health services on behalf of certain groups or entire populations” [7]. In order to be strategic, purchasing needs to align funding (and incentives) with legal entitlements of populations to health services, and allocations need to be linked, at least in part, to information on aspects of provider performance and the health needs of the population served [7]. Becoming more strategic as a purchaser involves movement along a spectrum. Meessen^1^ stresses how purchasing aims for an efficient allocation of resources to producers of (equitable) good health, and focuses on the role of purchasers and of stewards (e.g., Ministries of Health). In order to be strategic, purchasers should focus on (i) identifying the best value, (ii) selecting the right providers, (iii) designing smart contracts, (iv) efficiently enforcing such contracts. Under this, the role of provider payment mechanisms and the best use of information (through data, digital solutions and learning - [8]) are deemed to be essential to support strategic purchasing. The potential for strategic purchasing to also reduce fragmentation is acknowledged^i^.

Others have conceptualised (strategic) purchasing as involving three sets of decisions: (i) identifying the services to be purchased, taking into account population needs, national priorities and cost-effectiveness; (ii) choosing service providers, giving consideration to quality, efficiency and equity; (iii) determining how services will be purchased, including contractual arrangements and provider payment mechanisms [9]. These three sets of decision in turn require purchasers to engage in three main relationships, with governments, with citizens and with providers. For the purpose of this paper, we have selected an analytic framework derived from this last definition [9], which unpacks the concept of strategic purchasing under those three main decisions and relations (Table 2). Despite the differences in definition and conceptualisation of strategic purchasing, there is agreement on the fact that it is a core element of every health financing system and is essential to ensure progress towards UHC as it has the potential to ensure improvements in the efficient use of funds and in terms of quality of services and equity [10, 11].

This article sets out to start filling the gap in empirical analysis of the interaction of RBF and strategic purchasing by examining the case study of Zimbabwe, which since 2011 has gradually rolled out a national RBF programme focused on maternal and child health (MCH) indicators. Zimbabwe experienced a severe economic and social crisis, which peaked between 2005 and 2008 [12]. The crisis was characterised by dramatic reductions in the value of funds allocated to health facilities and health offices. Mitigation measures undertaken by government and development partners included various forms of targeted funding for core health programmes, including human resources retention, MCH services, and procurement and distribution of essential health commodities.

RBF started in July 2011 [13] as another response to the health sector crisis. Initially, RBF started in two front-runner districts of Marondera and Zvishavane, then in an additional 16 districts, with funding from the World Bank and implemented by Cordaid. RBF was scaled up to all rural facilities in 2014, with funding for the other districts coming from the pooled Health Transition (later Development) Fund (HDF), implemented by Crown Agents. A World Bank impact evaluation has been conducted [14].

In this article, we do not focus on the effectiveness of RBF, but we examine its role as a strategic purchasing mechanism, reflecting not just on the international debate on this topic but also how the introduction of RBF was framed in Zimbabwe, at least by some actors [13]. To do so, we look at the purchasing arrangements pre-crisis in Zimbabwe and the changes introduced through the RBF programme, and draw lessons on the potential of RBF as a strategic purchasing intervention in similar contexts.

## Methods

### Study design

The case study is largely retrospective, focusing on the period since 2011, although drawing on insights into the health system in Zimbabwe pre-crisis from earlier studies [15, 16]. It is based on KI interviews at national, provincial and district levels, integrated with analysis of documentation (policies and strategies, project documents and manuals, project evaluations and academic articles).

### Study sites

Data collection was done at national level and in two provinces (Midlands and Mashonaland East), including four districts (Murewa, Marondera, Gokwe North and Gokwe South). These provinces were selected as they were the sites for the pilot districts in 2011. The districts were chosen as representing one each from the two schemes (Cordaid and Crown Agents) per province and including the two pilot districts.

### Document review

We searched for documents on RBF in Zimbabwe from sources such as reliable websites (both for peer-reviewed and grey literature, including the World-Bank RBF website, the PBF Community of Practice and government websites), suggestions from KIs from government departments, donors and NGOs (including the implementers, Cordaid and Crown Agents), as well as documents already available because of the long term engagement in-country of the researchers. The documents included the following:

- National health strategic plans and health financing and system policy documents
- RBF project implementation manuals
- RBF evaluations and reports
- Minutes and reports from RBF meetings and working group discussions
- Relevant academic articles

A snowball technique was adopted by checking the references provided in the documents analysed and retrieving further relevant documents.

The documents date from the decade after 2008 – i.e. after the most acute period of crisis and prior to the introduction of RBF in 2011 – up till 2018. Some 60 documents were reviewed, the vast majority of which were operational and grey literature.

### KI interviews

Purposive sampling was used to identify KIs at national, provincial and district levels, based on their knowledge and involvement on RBF from its inception. The selection of interviewees was as comprehensive as possible, including individuals currently holding RBF-related posts or who were previously in such positions. A number of relevant organizations, groups and individuals involved in RBF were preliminarily identified. New individuals were added based on the results of the documentary review or as suggested by KIs. Individuals to be interviewed included representatives of:

- Ministry of Health and Child Care (MoHCC) – departments at central level, but also Provincial Health Executives (PHEs) and District Health Executives (DHEs)
- Other relevant ministries and national organisations, such as Ministry of Local Government and Rural Development and the Zimbabwe Association of Church-related Hospitals (ZACH)
- Development partners and RBF funders present and past: World Bank, HDF, Department for International Development (UKAid), the European Union, and United Nations agencies (such as UNICEF)
- RBF implementers: Cordaid and Crown Agents, at central and district levels
- Consultants and technical assistants who had worked on RBF

The breakdown of KIs interviewed (40 in total) is provided in Table 1. 18 MoHCC staff were interviewed at national, provincial and district levels. The development partner group was the next-largest constituency, with 10 KIs. Overall, men predominated, reflecting gender discrepancies in public service, particularly at higher levels. For RBF implementers, by contrast, staff at central and field offices were more commonly female.

**Table 1:** KIs summary

	Male	Female	Total
National MoHCC staff	5	0	5
PHEs	3	3	6
DHEs	4	3	7
Other ministries and public bodies	1	2	3
Development partners	7	3	10
Consultants	3	0	3
Implementers	1	5	6
TOTAL	24	16	40

KIs were approached by email or telephone, providing them with a brief explanation of the research project. A time and date for an interview was agreed upon. Before the interview, the researcher explained the study objectives and scope, and informed consent was obtained in writing. Confidentiality was assured. Consent was requested for recording, with manual note-taking as a fall-back option where the respondent was not comfortable with the conversation being recorded or where security arrangements or technology did not permit recording. 26 out of 40 interviews were recorded.

KIs were interviewed in English, using a semi-structured interview guide, based on the strategic purchasing framework [9], using a topic guide which was developed by the research team (supplementary file 1). Most interviews took place in the informant’s place of work, but in a location where privacy was assured. Some interviews were conducted by phone or Skype, where physical distance or access necessitated it. Interviews focused on the period from 2008 (prior to RBF introduction) to present and were tailored to the time available and the knowledge of the KI. Interviews lasted from 30 min to 2 hours, with an average of 1 hour. The questioning was led by a senior researcher, with a colleague assisting in taking notes. Interviews took place from early February to late March 2018.

### Analytic framework

We analyse our findings using a strategic purchasing framework which has been adapted from the literature [9]. The framework reflects the conceptualisation of strategic purchasing as the interaction between the purchaser and three levels of stakeholders: governments, citizens (or the population) and providers. The framework provides a descriptive and comprehensive list of actions and decisions that need to be made with reference to these three sets of stakeholders to ensure (strategic) purchasing (Table 2). The framework was chosen to provide a clear, pre-existing conceptualisation of a broad concept. We later reflect on the advantages and disadvantages of the framework chosen.

**Table 2:** Key actions for strategic purchasing in relation to different stakeholders within the health system

Key strategic purchasing actions by *government*	•Mobilising resources to meet service entitlements •Holding purchasers accountable •Assessing service delivery infrastructure gaps
Key strategic purchasing actions in relation to *citizens/population served*	•Ensure community participation and engagement •Assess needs, preferences, values of the population and use them to specify benefits
Key strategic purchasing actions in relation to *providers*	•Establish service agreements/contracts, and accredit providers •Develop formularies and standard treatment guidelines •Design, implement and modify provider payment methods to encourage efficiency and service quality, establish provider payment rates and pay providers regularly •Allocate resources equitably across areas and implement other strategies to promote equitable access to services •Establish and monitor user fee policies •Secure information on services provided/ develop, manage and use information systems •Monitor provider performance and act on poor performance •Audit provider claims and protect against fraud and corruption

The choice of an existing framework was done explicitly to avoid creating something ‘ad hoc’ for our analysis but rather rely on a previous, theoretical exploration of the concept. However, given the novelty of the concept of ‘strategic purchasing’ and its operationalisation, we recognise that there is a discussion to be had around the framework itself, beyond our specific findings. While this is not the purpose of the paper, we briefly do so in the first part of the discussion section.

### Data analysis

Data analysis was done iteratively. A first analysis of the documents collected was conducted before the interviews in the field, and guided the discussion during interviews. Later on, new documents were added to the review, and a final thematic analysis [17] was conducted of documents and interview transcriptions or notes, using mostly pre-defined themes based on the strategic purchasing framework [9] which was adapted to the specific context and to reflect on the role of fragility in the case study (Table 2). Results of the analysis of documents and interviews were written-up together to allow for triangulation and complementarity between data sources.

### Ethics

Ethical clearance was obtained from the Research Ethics Panel of Queen Margaret University, Edinburgh, and from the Medical Research Council of Zimbabwe. The study also received authorisation from the MoHCC.

## Results

Based on the analytical framework adopted, our analysis considered in turn which bodies were undertaking purchasing functions and actions (as listed in the framework) prior to RBF, how clear their roles were and how these changed (or did not change) as a result of the introduction of RBF in Zimbabwe. The recent history of the health system and the political-economic crisis in Zimbabwe is important both as a catalyst to the introduction of RBF but also in understanding the legacy of capacity within which RBF operated [13].

## Key strategic purchasing actions by government

The key purchasing functions at government level are considered to include: mobilising resources to meet service entitlements; holding purchasers accountable; and assessing service delivery infrastructure gaps [9].

### Mobilisation of resources to meet service entitlements

Since independence, the public health system in Zimbabwe was developed as an integrated model, with planning starting at rural health centre (RHC) and district level, which was coordinated through the Provincial Medical Directors (PMDs) and fed into national budgets. Resources to support the provision of the primary care package (established in 1995 [18] were mobilised through central taxes, with additional revenues at the local level from user fees.

The growing political-economic crisis, which culminated in hyper-inflation in 2008, led to a collapse in private and public resources for health care [19], and facilities were forced to charge the public for services, even those which were meant to be free, such as MCH. The public and private sectors entered survival modes – hardly able to pay their staff and to continue to function. At this time, those with diverse sources of funding (such as municipalities, which are able to collect local taxes) were better able to reward and keep staff [16, 20]. After the height of the crisis in 2008, the role of donors increased, providing resources in the recovery period after the formation of the Government of National Unity in 2009. Resources were employed to re-attract and retain staff, purchasing essential supplies and providing technical and material inputs to improve the quality of care. Donors’ support was focused on the primary level and especially maternal health, as maternal mortality rates rose dramatically during the crisis [21].

Since its introduction in 2011–12, RBF has provided an estimated extra $2 per capita amount, focused on core MCH indicators, along with one indicator targeting new outpatient cases.^2^ RBF has been supported by the Health Development Fund (a pooled donor funding mechanism), the World Bank and the Ministry of Finance, which provided a small co-financing to the World Bank-supported programme since 2015. The World Bank funds could be regarded as additional, as the funding was conditional on Zimbabwe adopting a result-based financing modality. On the other hand, the HDF funding was already being invested in an input-based manner in 2011–14 (under its earlier label of Health Transition Fund), and would likely have continued as such if it had not shifted to RBF in 2014 for part of its support. Both funding streams have been reducing and are insecure in the long term [22].

Despite the donors’ funding, and given the on-going economic and fiscal challenges in Zimbabwe, overall resource mobilisation by government (and donors) for purchasing health care services remains inadequate [23], with households contributing around 25% of total health expenditures in 2015, of which 95% out of pocket [24]. As a result of the lack of funding, purchasing is at times perceived as an empty exercise.

‘*Purchasing is very under-developed because there are no public funds. It boils down to purchase of basic commodities’* (national KI)

### Accountability of purchasers

Different purchasers account to different constituencies – the Rural District Councils (RDCs) report to the Ministry of Local Government and local populations in relation to rural primary health care, while urban primary care and some public health services are purchased by municipalities (also under the Ministry of Local Government). The MoHCC accounts to the Ministry of Finance and Economic Development, as well as downward through health facility committees to communities.

Two parastatals were also established to purchase and coordinate specific services - the National AIDS Council, established in 1999 to administer the National Aids Levy funds and purchase HIV services, and the Zimbabwe National Family Planning Council, which leads family planning purchasing and service delivery in Zimbabwe [18]. They have integrated performance agreements with the MoHCC.

Mission health facilities have over the years received grants from the MoHCC but there is still no clear Memorandum of Understanding or formal service contracts between them [18]. As a consequence, there has been very limited oversight and accountability around the disbursed grants to mission facilities. This reflects in part limited capacity within the MoHCC to monitor the performance of mission facilities receiving these grants [25].

During the crisis, most of these accountability systems had stopped functioning because of the lack of resources for purchasing. In addition, development partner funds could not be channelled through the government system, given the poor relationships with then Mugabe-led government. As a result, the bulk of aid funds are managed by a few external organisations – for example, UNICEF is the fund-manager for the HDF, and manages medicines, supplies and commodities procurement, while the United Nations Development Programme is the main Principal Recipient for the Global Fund for AIDS, TB and Malaria. There is limited transparency in relation to their overhead costs and overall expenditure, as well as the familiar challenges of fragmentation and lack of coordination.

Within the introduction of the RBF programme, new actors were entrusted with the some parts of the purchasing role. For MCH services at primary level in particular, the two external implementation contractors, Cordaid and Crown Agents, play a lead role in purchasing, although they are guided by a national steering committee to which they are accountable.

### Service delivery infrastructure

Infrastructure is managed by a range of bodies, including the mission sector, municipalities (which manage urban health facilities) and RDCs, which own the majority of rural primary care facilities. The MoHCC exercises overall stewardship and is required to give permission for new (public) infrastructure. An assessment of RBF carried out in 2016–17 suggested that rationalisation of infrastructure was needed – it noted that even the most effective purchasing methods driven through an inefficient provider system will produce sub-optimal results [22]. Density of population and catchment populations vary significantly (e.g. from 2000 to 11,000 people per health centre in just one district visited), making it hard for facilities with smaller populations (often more remote ones) to generate funds and bonuses. With RBF, the flow of funds follows service use, which tends to reflect the existing infrastructure.

*‘A scheme like this tends to solidify existing infrastructure. It does not distinguish between efficient or not. It doesn’t tend to drive mergers or networks of providers, for example’ (national KI).*

There is no evidence that RBF in Zimbabwe has promoted a review of infrastructure arrangements. The mixed ownership of health care infrastructure and low budgets for capital expenditure, as well as political factors, make reforms to infrastructure challenging.

## Key strategic purchasing actions in relation to population served

In this section, we consider purchasing in relation to, first, community participation and engagement, and, secondly, in relation to assessing the service needs, preferences and values of the population and using them to specify service entitlements.

### Community participation and engagement

Community participation and engagement is included in the analytic framework we adopted as an essential element in strategic purchasing. It includes informing the population of their entitlements and obligations, ensuring the population can access their entitlements, establishing effective mechanisms to receive and respond to complaints and feedback from the population, and publically reporting on resource use and other measures of results [9].

Zimbabwe had a history of strong community engagement in health care [26]. The Health Services Act of 2004 established health facility committees at various levels, and a Patient Charter was introduced in 1996. However, only 49% of health centre committees (HCCs) sampled were still functioning in 2010, after the economic crisis [26], when a survey found evidence of wider but limited in-kind contributions, such as labour, from communities to health facilities, particularly in rural areas.

One of the conditions for being contracted under the RBF programme was to have an operational HCC [27], and the HCCs have been incentivised to be more active as the health centres now have resources to manage directly, in line with the operational plans which they have to develop. As with HCCs, operational planning pre-dated RBF but has been given a boost by it as resources are now available to carry out what were previously theoretical plans. 86% of HCCs surveyed in 2016 were functional [28]. The Health Transition Fund evaluation finds variable performance across the HCCs, linked to local dynamics, but concludes that community links were probably stronger than before the programme. An evaluation of an independent project to support HCCs [29] concludes that RBF led to a change in HCC roles, which had previously focused on resource mobilisation and now focus on resource allocation. In addition to revitalising the role of HCCs, RBF enforces a number of elements of community accountability, such as provision of suggestion boxes and making pricing visible in facilities as part of its quality checklists. Verification also includes quarterly client satisfaction surveys by community-based organisations or the implementing agency field officers.

Despite these processes linked to the introduction of RBF, community members showed low awareness of the work of the HCCs and reluctance to use mechanisms such as complaints boxes [28]. The World Bank impact evaluation also highlights problems of political interference, low capacity -especially on financial management as members are drawn from the community and may have no accounting background - and lack of representativeness of HCCs [14]. The evaluation concludes that they are unable to offer meaningful community participation, or to improve the quality and utilisation of MCH services. At the same time, the client satisfaction surveys appear rather insensitive with scores above 80% on average and unvarying over time [22]. Additionally, it is not clear how well the results of those surveys are fed back into improved responsiveness of services.

‘*There is now some measurement of client satisfaction and some feedback. However, we need improvement in how we respond to these. For example, the HCC should be discussing the scores’* (national KI).

### Assessing service needs, preferences and values to specify service entitlements

Despite the efforts described above to strengthen community participation and engagement, there has been no consultation on the users’ needs or preferences to feed into the benefits package, as this is agreed nationally and there is no scope for variation at local level.

## Key strategic purchasing actions in relation to providers

In this section, we examine how providers are contracted and paid, how the crisis impacted these purchasing functions by starving them of resources, and how these functions have been affected by RBF.

### Establishing service agreements/contracts and accrediting providers

Some elements of active purchasing and contracting existed in the Zimbabwe health system before the introduction of RBF. For example, in 2005 a Results-Based Management approach was introduced into the health system, but it was never fully operationalized due to lack of national funds [30] [13]. A literature review of health care purchasing in Zimbabwe [18] highlighted the lack of clarity in contracts and entitlements across the other sectors (e.g. local government and the private sector), as well as in finance for these from various subsidy streams. It commented on the lack of separation of regulation and purchasing functions in the MoHCC, Ministry of Local Government and mission sectors. Performance agreements existed between the levels of the MoHCC but without supportive resourcing, while these are lacking with or within local government and the private not-for-profit sectors. Private health insurance and private social insurance were found to be spending more on investment and administration than direct service provision.

RBF reintroduced a contractual approach, with eight contracts being specified by Zimbabwe RBF Project Implementation Manual [27], one of which was with providers.

In terms of providers’ accreditation, RBF has not introduced any changes to the existing system – all public and mission facilities were accepted within RBF as long as they met the minimum managerial criteria, such as development of operational plans, having a functional HCC, having a bank account, and agreeing to remove user fees for mother and child health care (a prior MoHCC policy which RBF aimed to reinforce).

*‘The World Bank wanted institutions to qualify to be included. We wanted all in – if they were weak, they could be supported to improve’ (national KI).*

### Developing formularies and standard treatment guidelines

Equally, RBF has worked within existing clinical guidelines, which were relatively well developed prior to the crisis. The health system assessment of 2010 found well developed guidelines for treatment of common conditions, though their availability at facility level was mixed [26].

### Designing, implementing and modifying provider payment methods to encourage efficiency and service quality; establishing provider payment rates and paying providers regularly

Historically, much of the purchasing was done at national and district levels. Funds from central level and user fees were retained by hospitals, while virtual accounts were held at district level for the purchasing of commodities on behalf of the primary health units. Medicines and supplies were nationally purchased and supplied using a pull system. Staff were hired centrally, deployed by the provinces to facilities, and paid salaries from the national payroll. Purchasing of public and private health services through health insurance, including medical aid for civil servants, covered around 10% of the market, with contracts specifying price and quality.

The introduction of RBF did not affect the purchasing arrangements in the wider public sector and the purchasing arrangements and payment mechanisms remain mixed. The bulk of public resources continue to go to staffing through salaries - in 2017, salaries made up around 79% of total MoHCC allocations and a much higher share of total actual expenditure [24] – as well as medicines and supplies, through input-based channels. Resources are still managed at district level for non-RBF funds; these include the Health Service Fund (for user fees and donated funds), and Government of Zimbabwe funds, with shadow budgets operated at district level on behalf of primary facilities. Within the MoHCC, individual programmes also undertake purchasing for their specific commodity areas, adding to the under-powered and fragmented public purchasing system. A number of the main health system pillars also have their own agencies to purchase inputs, with the Health Service Board hiring and managing health staff, and NatPharm the medicines and supplies. The private health insurance market (consisting of some 40 medical aid societies) continues to function but is squeezed between rising costs and supply-induced demand, on the one side, and limited ability of the population to pay for health insurance on the other [25]. In theory, providers should operate fixed tariffs for insured patients across the industry but these are reportedly not respected.

At the facility level, historically, facility budgets were based on a mix of past trends and planned activities, though half of the District Medical Officers and the bulk of PMDs surveyed in 2010 reported using results-based budgeting [26]. RBF changed the payment mechanism in particular for primary care units, as discuss below in relation to (i) design, (ii) effects on quality of care and (iii) efficiency.

#### Design issues & regularity of payments

RBF has focused on maternal health, reflecting priorities post-crisis. The payments per indicator are based on a number of factors, including the available budget (which appears to be the dominant element to influence payments), levels of desired coverage, which indicators were lagging, and stipulated distribution between primary and secondary level facilities. The ratio has been fixed at 60% (of total budget) to primary and 40% to secondary, though the rationale behind this ratio is not clear [31] [28]. However, the payments per indicator do not reflect actual production costs for services nor were they pegged to previous fees charged by facilities. As one of the respondent said,

‘*It is not very strategic. Prices [for RBF indicators] are set by the budgets, not the other way round. And the budget is also never enough’* (national KI).

A drop in prices in 2013 led to overall fall in facility revenues, with 63% receiving below the budgeted amount per facility. Analysis by Cordaid does not however find any impact on the performance of indicators of this price reduction [32]. This indicates that RBF may be functioning less as an incentive and more as a useful general source of resources at primary level to enable providers to do their work. It also found a high correlation between earnings and the catchment population of the facility, suggesting that size of catchment population is the main driver of earnings [23, 33].

In addition to RBF, facilities have continued to receive other sources of support, not least for their main expenditures, such as staff and medicines. However, RBF augmented these and was significant in bringing flexible funding to the RHC level at a time of national budget shortfalls.

*‘We noticed positive changes. But it was not just $15million buying results. The HTF was bringing in essential good [s], and money for meetings etc. RBF helped make use of HTF resources efficient. So it was a combination of input- and output-based payments. It wouldn’t have worked with just one. We needed everything’ (national KI).*

There is therefore concern that the budget and payments drop due to funding shortfalls in 2017–18, alongside inflation in general costs, will undermine gains. There was a reported 60% drop in facility revenues in late 2017, completely unconnected to any change in performance. Caesareans, for example, which were paid $140 previously (not enough to cover costs, according to some), are now only paid $40.

*‘RBF works through financing. If it is underfunded, then it does not work … If funding goes, it will be hard to deliver these services’ (local KI).*

The hospitals have also been starved of funding, especially in the HDF districts where hospitals received flat rate subsidies [22]. In the referral hospitals supported through the World Bank, RBF was estimated to provide 80% of their income, even though it only covered a few indicators [22]. This is being addressed in 2018 through extending RBF contracts to all district hospitals as well as an increased national budget allocation for hospitals.

Encouragingly, there is evidence of learning and iteration in the design of the RBF programme – for example, adjustments which were made in 2017 after the health system assessment [34]. These revisions were agreed through a participatory Delphi process with subsequent adjustments. However, adjustments are complex, as they have to take into account trends in coverage of indicators, changing burden of disease and strategic priorities, alongside a limit on the budget. Currently, indicators are fixed nationally but there is some discussion of allowing for more local tailoring to health priorities.

In terms of regularity of payments, all payment systems have been affected by the financial crisis in Zimbabwe, causing delays and shortfalls. RBF has provided financial relief for facilities but is itself now subject to budget pressures, leading to reduced payments and also delays of several months in paying providers.

#### Quality of care

Quality of care attracts additional payments in the Zimbabwe RBF programme. A quarterly checklist is filled by the DHE (for RHCs) and the PMDs (for district hospitals). The maximum weight for quality of care was 25% of overall payments, though in practice only 18% was disbursed for quality in the Cordaid scheme [34]. As outputs are now high, but health outcomes still lagging in some key areas, the 2016 assessment recommended an increased weight for quality of care [22]. In addition, staff bonuses are now tied more closely to the quality score, as this is seen as more clearly within their influence; for those receiving less than 60% on the quality index, all staff bonuses are lost. The aim was also to ensure that populations are not penalised (through reduced resources) for poor staff performance [22]. This change has helped low catchment facilities, but penalised high-volume ones (which used to be able to cover their costs through the higher quantity payments).

The original quality checklist was very oriented to reproductive and MCH services but has now been expanded to be broader and also to focus less on structural factors and more on clinical (the weighting of the scores is now 65% clinical, and 35% structural, as adjusted in 2016). It now includes a number of domains - structural; management and planning; client management; and client satisfaction - with on-going debate about their appropriate relative weights [34]. The community satisfaction survey results feed into it and contribute 20% of the overall quality scores.

In the impact evaluation, client satisfaction with antenatal services in RBF facilities was found to be higher overall, but there was no significant difference for child health services. Few significant differences in structural or equipment indicators were found, though there was some evidence of improved medicines availability [14]. Out of eight quality of care domains, only one (family and child health) showed significant improvement in RBF facilities over controls.

Quality scores are high and have plateaued (all falling between 84 and 87% in the 42 HDF districts in 2016 [22], so it is not easy to discriminate on quality. These scores suggest either that performance is genuinely high across almost all facilities or that the measurement process is not sufficiently sensitive or robust. As the quality scoring is carried out by the DHEs, it would be understandable if there was internalised pressure to score highly. It also remains important to use the tools to guide supportive discussions to identify and address problems.

‘*We should be diagnostic about why quality scores are lower [when they are]. There is usually a conversation about this but limited follow up, which is demotivating. We need to focus on carrots more than sticks’* (national KI).

The RBF programme has coordinated its approach with the wider national strategy on quality improvement [35], and has benefited from many wider investments in quality – for example, the up-skilling of Primary Care Nurses, and projects such as the MCH Integrated Health Programme, which provided training in obstetric care. However, concerns remain on the length of time taken to fill in quality assessment checklists - an estimated 2–3 h per RHC, occupying the nurse who also is lead clinician in the facility. There is an overall need to reduce duplicating data collection (for example, the President’s Emergency Plan for AIDS Relief checklist is reported to take 6 hours to fill in and have overlapping questions).

#### Efficiency

In terms of outputs, the impact evaluation of the 18 district RBF scheme in Zimbabwe showed some positive results [14, 35], although it should be noted that resources were not matched across intervention and control sites (so some of the results may follow from increased resources rather than RBF as a payment mechanism). In a period in which there was a general recovery in MCH indicators throughout the country, faster improvements were made in the 18 districts in institutional births and post-natal care. This differential did not apply to antenatal care attendance, modern contraceptive use, and immunization rates. There was no evidence of consequential neglect of non-incentivised services.

Some of the RBF indicators are high and appear to have reached a threshold beyond which it is hard to go (leaving residual hard to reach populations, especially those with cultural barriers). Others continue to under-perform – for example, postnatal care (which had a coverage of 47% in 2016) and supervised deliveries (59% coverage in 2016) [22]. Some community-based services, like vitamin A distribution, continue to be under-reported. This highlights the need for continual adjustment of indicators to ensure that purchasing is indeed strategic.

As RBF pays per output, with added bonuses for quality, it could be seen as inefficient in a system with motivated providers (who are being paid to deliver results that they would anyway have strived to deliver). In this context, incentivising them to reach the final 10–20% hard-to-reach populations would make more sense. However, in Zimbabwe, the crash in national budgets has meant that RBF is covering basic running costs. It has enabled reasonably flexible resources to reach the front-line providers, which most informants saw as an important improvement given the post-crisis constraints.

The incentivised indicators include services with high coverage, such as antenatal care, while others with high burdens of disease have not been included. A recent report pointed out that there was no tuberculosis indicator, for example, while this is the fourth cause of disability-adjusted life years lost among women of childbearing age [36] [32]. This has been addressed in 2018 through the addition of some wider indicators, though the payments attached are currently so low as to be demotivating.

Given the complexity of RBF and the separation of functions, the programmes have incurred high overheads, running at up to 23% of scheme costs over and above the existing management costs of districts, though it is acknowledged that these have reduced over time and include some important elements such as training and quality improvement [22]. Some of the tools are also costly to administer – the cost of the community satisfaction survey, for example was found to be US$ 5 per questionnaire, which was much higher than the additional subsidies allocated to health facilities from this component [32, 36].

Another concern has centred on the inefficiency of medicines being purchased at facility level, compared to the previous pull system (pre-2009), when medicines were centrally procured. District hospitals were found to be spending around 26% of their RBF funds on medicines in the Crown Agents districts [22], but with higher levels at RHCs. Health facilities can buy from approved pharmacies but there are no long term pricing agreements as yet to help reduce medicine costs. This however is a consequence of wider problems, unrelated to RBF: the central agency, Natpharm, remains extremely underfunded and 99% of all the pharmaceuticals handled by it are donor-funded [24]. The need for coordination of various financing streams in procurement and distribution systems for commodities is widely recognised and reforms are being proposed [22].

There has been limited analysis of how RBF funds have been spent. In the initial phase, there was a focus on infrastructure, especially the construction of maternity waiting homes, though it is unclear how well used these now are.

*‘It is still not clear how the facility funds were used. It is hard to assess purchasing at the facility level without that data. Is procurement more expensive at the facility level? It is still not clear’ (national KI).*

The MoHCC recently sent instruction to limit expenditure on travel and subsistence to 10% of RBF revenues, indicating a concern about the costs of purchasing, raising quotations and so on. A move to electronic quotes and payments would reduce these costs but with poor network access, this may be a challenge for many RHCs.

### Allocating resources equitably across areas, and implementing other strategies to promote equitable access to services

Zimbabwe has focused on equitable health system development post-Independence. However, it lacks a resource allocation formula – this is highlighted as a priority in the Health Financing Strategy [25]. Public budgets have been set based on historical patterns and planned activities. With RBF, funds are not allocated by area but follow utilisation, which benefits facilities with large catchment populations (which are often in better connected, and therefore more prosperous, areas). There were concerns early on about RBF reinforcing inequities in the system, so there was an initial infusion of funds for all facilities to help them reach a minimum standard. However, preliminary calculations in 2016 indicated that even with additions for the quality score and remoteness payment, about 60% of RHCs earn less than $600 per month, which is judged to be the minimum operating cost of the average health centre [22]. Such facilities are also likely to be adversely affected by the application of the 5% inaccuracy rule (which penalised inaccurate reporting). They were unevenly distributed across provinces, with some provinces, such as Matabeleland, suffering from low RBF earnings in particular [23, 33].

Some clinics in towns were included after the start of RBF in order to stop the flow of clients from towns to RHCs, however most urban areas and the two main cities, Harare and Bulawayo, are excluded. (There was a voucher programme piloted in these two areas^3^ but this was small-scale and limited in terms of services covered.) This is problematic for the deprived areas within them. An urban delivery voucher scheme has been piloted but remains low in coverage.

While infrastructure is distributed relatively equitably in Zimbabwe, staffing is less so, especially for doctors [37]. While RBF does not directly influence this, it may have incentivised some staff to move from district hospitals to RHCs, especially in areas where RHCs were funded through RBF and district hospitals through flat-rate subsidies (as the staff incentives were better at the RHC level in these areas). This will presumably reduce in 2018 as district hospitals join the RBF programme.

RBF’s focus on MCH care at the primary level is inherently equitable, and the programme incorporates additional payments to allow for facility remoteness [27]. Depending on the level of “remoteness”, an additional payment is made to the facility of up to 30% of the value of the quantity payment [22], though in practice in the Cordaid Scheme 4% of the total payments were made based on remoteness [34]. This is unlikely to provide sufficient incentive to reach challenging populations.

### Establishing and monitoring user payment policies

Zimbabwe has offered MCH services at primary level without user fees since the 1990s [37]. At secondary care level, mothers and children and those over 65 years of age do not pay for services. However, during the crisis years, these policies were not fully enforced due to lack of financing for services and lack of supplies and medicines [18]. RBF and other investments, like the HDF input-based financing, helped to enforce the policy.

RDCs previously collected revenues from their health facilities [37] and there was some reluctance to stop this source of fund-raising. Mission facilities were also dependent on user fees. The RBF programme helped to enforce the removal of user fees for MCH services across these sectors (though not in municipal facilities, which are in urban areas), and also to negotiate that RBF payments were retained and managed by health facilities. Some RDCs are reported to be continuing to levy small charges, which are used to pay for security guards. However, one report notes progress from 60% of facilities offering fully free maternity services in early 2012 to 95% in 2015, with RBF facilities more likely not to charge fees [28]. By late 2017, only 2.4% of hospitals and 10% of secondary or higher facilities were reported to be charging for antenatal care, one of the core free services [38]. The RBF impact evaluation, however, does not find a lower incidence of out of pocket payments in RBF versus control facilities [14].

Zimbabwe used to operate a social welfare system in which Assisted Medical Treatment Orders, managed by the Ministry of Labour and Social Services, were used to identify vulnerable people for exemptions from user fees. This system broke down due to lack of funding during the crisis. In the RBF facilities, HCCs are meant to identify people without access and support them, but this may not be very systematic. User fees remain a burden for all other services, including at hospital level, for other uncovered services and sectors, and in urban areas, contributing 24% of overall health expenditure [25]. Other mechanisms are needed to support financial access, and to address cultural barriers (for example, in the substantial sub-population which reject medical interventions for religious reasons).

### Securing information on services provided, and developing, managing and using information systems

The Health Management Information System (HMIS) was reported by some informants to be weak pre-RBF, with little incentive to focus on accurate reporting at the facility level. However, an assessment in 2010 suggests that the system was better than in many sub-Saharan African countries, with 80% completeness, 68% accuracy and 67% timeliness for the main T5 form (which reports on outpatient care at district level), though capturing only an estimated 50% of private sector services [26, 39]. Many services, like growth monitoring, were provided but not recorded as that was an additional task. Feedback to districts and provinces was good, but less systematic at facility level (49% of facilities reported receiving written feedback on their data in 2010 [26].

RBF has not influenced the reporting (or lack of it) by the private sector but is seen by many as having placed a helpful emphasis on quality of reporting by public, predominantly primary level facilities. Under its rules, over- or under-reporting of indicators by more than 5% results in no payments being made for that indicator. This is seen by some as too draconian: this is a normal margin of error and penalises facilities (which have incurred expenses for the services provided) for what may be an error of just one case at the facility level. Most report fewer than 20 outputs per indicator per month [40] [36], so with only one incorrectly recorded in a minor way may lose the entire funding of that indicator. The estimated lost income from this rule for the 42 Crown Agents districts was $3.7 million over two years (2014–16), which meant considerable resources not invested in improving maternal, newborn and child health [36, 40] – some 46% of the resources actually paid out.

*‘It is fine to adjust for quality, but they shouldn’t be paid zero when they have forgotten to put the telephone number of the lady who was delivered. If we did this in the UK, for example, there would be an outcry!’ (national KI).*

For the Cordaid scheme, however, the average percentage loss of subsidies dropped from 50% in 2012 to 7% by 2015, allowing for the introduction of a lighter risk-based verification system.

Overall, there is an understanding that the gap in reported and verified data has closed over time. This is widely seen as one of the main benefits of RBF, but may be partly an artefact of measurement – aggregate data over a quarter is presented, in which under-reporting by some facilities is balanced out by over-reporting in others, thus making reported data look close to verified. On a facility by facility basis, however, more than 50% of facilities are still having errors, even on simple indicators like antenatal care visits, even after 3–5 years of RBF implementation and support. Analysis of data for the Crown Agents facilities in 2016 found that lost income from the 5% error rule stabilised at 27–29%, suggesting that 2 years into implementation, facilities were still losing considerable amounts due to poor data [40, 36]. The HTF evaluation confirmed the complexity of accurate reporting, as perceived by health staff, even after several trainings [28].

*‘We should be progressing but are staying in the same place, as we continue having to retrain on the basics, like data entry’ (local KI).*

At facility level, staff continue to face the problem of multiple data reporting and data streams – RBF is not responsible for this, however, it has not helped to reduce the burden. There are 34 registers to be filled [41] [21], as well as overlapping surveys which absorb staff time.

*‘Beside the HMIS and the national data warehouse, stakeholders are conducting several parallel routine health facility evaluations processes. For the time being, each rural health care facility is surveyed at least 12 times a year (this does not take into account HIV, TB, malaria, supply chain supervisions and SARA surveys)’ (national KI).*

*‘The staff are sometimes overwhelmed. They have to balance the day-to-day running of the facilities and the quality of data … Many who were trained in 2014 have now left and so there is a need for continuous monitoring and training’ (local KI).*

There is a need to move to digital systems and merge data streams to make reporting and monitoring more manageable.

RBF implementers have been operating two RBF information systems, linked to the HMIS for quantity indicators and deriving data from it but not feeding back into it. However, in 2018 the Cordaid system migrated to the MoHCC as part of the institutionalisation programme. An integrated information system is appropriate in the long term but poses short-term risks.

In addition to better and lighter recording, correct analysis of data has been a challenge, given that the reporting system was based on HMIS data, which can be reviewed and revised retrospectively. This makes data management and trend analysis difficult, even for the implementation agencies.

*‘They are working in Excel files, with mistakes in relation to facility names, and therefore probably also in payments. … This is probably similar in other places’ (national KI).*

### Monitoring provider results and acting on poor results

Performance management systems were in place prior to RBF, but faced challenges in relation to their systematic application, including resource constraints, such as a lack of skilled personnel, competing priorities and high workloads, challenges in patient privacy and restrictive work spaces [18]. An assessment in 2010 found that most PMDs and District Medical Officers were reporting monthly or quarterly supervision, using an integrated checklist. Just under 60% of planned supervisions were carried out [26].

RBF has provided resources to support supervision at provincial and district levels. The Provincial Health Executives have four indicators, which focus on administrative tasks in relation to the RBF programme. DHEs have 12, which are a mix of performance-related and administrative. Some indicators are questionable – for example, staff posts being filled according to the facility establishment [27] is not within the control of the DHE, nor are the established posts up to date and therefore appropriate [22]. However, the funds provided through these indicators can enable wider support and supervision, if used well. There is now discussion of setting up national payment indicators for RBF supervision.

Previously supervisions were reported to be less clearly structured – focussed on high priority areas, like immunisation, but not comprehensive. This is seen as a positive spill-over for the health system of RBF, although, as noted above, there are concerns about the length of time taken to fill in quarterly supervision forms, especially for the DHE, which has to visit many clinics, often with difficult access, and which is penalised if reports are not timely. DHEs also receive support from other donors for supportive supervision and it is not clear if these funds are duplicating or are being used in an integrated way.

There are reports of inconsistencies in assessing too, such that scores may be higher in areas where there is less supervision, but lower where the checklist is used robustly. In these latter cases, there is more likely to be progress over time. District leadership and attitudes are a key factor here, as is committing time at the facility level and by supervisors to follow up on problems and action points.

‘*Performance varies by DHE. Everything works if you have a good DHE, whether input- or output-based. Some are good at motivating, and others not. The human factor is key*’ (national KI).

*‘The problems identified are often not resolved. A major reason is that the sisters have no time to go through the checklist when we have left. The health centres are burdened with 21 registers, each filled on a daily basis. The checklist is just another job for them’ (local KI).*

The facility operational plans, which existed previously but were less structured, are an important vehicle to identify and address priority issues which RBF reinforces. The facilities are also supported by the implementation agencies’ health field officers, who currently provide training, mentoring, verification (see below), and support for problem solving, especially on procurement at the facility. This raises concern about how to continue this level of support once RBF is institutionalised within the public system, as currently planned [42].

*‘The MoHCC does not have urgency … it is so overwhelmed... Will the facilities be paid on time? ..We need someone who is very accountable. The MoHCC is a regulator but not very accountable. It is responsive, not proactive … Many DMOs [District Medical Officers] don’t even know the checklist or where facilities are’ (local KI).*

RBF is embedded in wider national institutions. The district-level RBF steering committees are meant to meet quarterly and to report to the District Development Committees [27], but vary in their level of engagement. Normally, RBF issues are discussed at District Health Team meetings, which ensure intersectoral collaboration (with a similar structure at provincial level). As done pre-RBF, benchmarking is used to create additional performance pressures, with results shared in district and provincial meetings.

Wider rigidities remain within the health system – for example, constraints on recruitment due to the staffing freeze, the inability to remove under-performing staff and managers, resources which are channelled through vertical procurement programmes. RBF has not been able to influence these major inputs and processes in the health system. The RBF system itself is also somewhat unwieldy – given its complex systems, making alterations to reflect changing conditions and priorities is not simple or low-cost.

*‘RBF is very simple – it is just paying invoices at the end of the day. But with 1,000 facilities to train, the reality is that people don’t change the system easily. Once people have understood it, nobody dares to change anything. It is very expensive to do so too’ (national KI).*

### Auditing provider claims and protecting against fraud and corruption

Verification was not previously required as payments were not output-based. Under RBF, verification systems differ across the two implementers and have also changed over time. In the Cordaid programme, the local field officer originally provided the first-line verification, followed by external checks by the University of Zimbabwe until late 2017. The latter was dropped in order to save costs. The community sisters (based at the district) are now responsible for monthly verification in both schemes, though many report lack of access to vehicles and fuel to conduct these as regularly as expected. Counter-verification is then conducted by the Health Field Officers, who also conduct quarterly exit interviews to assess community satisfaction (in the Crown Agents districts). Community-based organisations are contracted to undertake these in Cordaid districts, to maintain more separation of functions. The plan for institutionalisation is that verification will be conducted by Community Sisters and HMIS officers (in hospitals), with counter-verification by members of the Health Profession Authority.

Overall, there is no evidence of significant gaming, which has allowed a shift to a risk-based verification system from 2014. In Crown Agents districts, only 2% of facilities are now considered to be ‘high risk’. This could be attributed to RBF controls but more plausible is that the system and organisational culture does not promote gaming.

In relation to finance and procurement, the DHE checks on procurement procedures, which are complex and follow national guidelines (there are, for example, 12 steps for RHCs to follow when procuring [27]. Each requisition requires three signatures, including by the HCC and DHE. In one district visited, about 30% of purchase orders are returned because of irregularities, mostly related to the difficulty of mastering the process and staff or HCC turn-over. Again, there are concerns about how these verification activities can be maintained in an integrated national system.

*‘It is an expensive mechanism because of the separation of functions. For example, an independent fund-holder – it costs to set up a separate entity … The verification is also heavy, you have to verify every little bit. With better IT, it should be easier, but we have limited capacity’ (national KI*).

A more mature system would likely use accreditation of facilities, electronic records and periodic clinical audits (as Zimbabwe used to have and which could be revitalised). While RBF has a strong focus on controls over proper use of funds and demonstrating success, some argue that it has been less successful in using data intelligently to understand problems in the system and how they might be addressed.

‘*PBF is now full of people crazy about control. This was not the original goal – it was to get money to the providers. … This doesn’t make sense any more. Control should not be the first purpose*’ (national KI).

## Discussion

This study has some limitations. In terms of data collection, care was taken to include all of the main stakeholders and participants in this policy, not just present but over its history. However, the interviews could not be comprehensive. In some cases, KI were time-limited so interviews had to focus on a limited range of questions. Equally, many of the documents which describe the process of policy development and roll out are confidential or not available, so while the researchers tried to access as broad a range of documents as possible, they could not be comprehensive.

More importantly, our analysis is based on an existing framework [9] which unpacks the concept of strategic purchasing based on the actions that needs to be carried out in relation to three sets of stakeholders. This framework is based on a broad and inclusive interpretation of the concept of ‘strategic purchasing’. As explained in the Introduction, others have suggested a narrower focus, focussing on elements such as identifying best values, accreditation and contracting of providers, as well as on the use of data^i^ .The breadth of the framework, which also gives equal value to each element, frames our conclusions on RBF’s impact on strategic purchasing and raises for further discussion the question of which elements are the most critical within it, and indeed raises the interesting question of how far RBF’s theory of change itself highlights the same or different elements as the strategic purchasing framework. This would be relevant for future investigation. Finally, it is important to note that the framework has been developed to analyse ‘strategic purchasing’ mechanisms in general and not specifically RBF or the effects of RBF on purchasing arrangements. Indeed, as elaborated below, RBF remains one of the mechanisms among others, and does not necessarily aim to cover all the actions and decisions of strategic purchasing – though arguably it should be aligned and integrated with the existing purchasing architecture^4^ .

Despite the limitations, this article adds to the limited literature on RBF in low and middle income countries and how it affects and makes more ‘strategic’ the existing purchasing arrangements. Based on our case study of Zimbabwe, it suggests that expectations of institutional reform and in particular the potential for RBF to drive a more strategic approach to purchasing [5] [4] should be moderated, particularly at early stages of RBF implementation [43] . Considering our strategic purchasing framework, we find (see summary in Table 3) that rather than systematically reforming strategic purchasing functions, in contexts like Zimbabwe’s RBF adds a new provider payment mechanism into the mix, which can produce benefits, but also adds to a complex landscape and does not resolve many underlying challenges [44] [45]^iv^. Some functions, such as assessing service infrastructure gaps, are unaffected by RBF, while others, such as mobilising resources are partially improved, as RBF in Africa has focused on one package of care (MCH services) within the wider essential health care, and even here has contributed only marginal costs. Purchasers’ accountability has typically been to funding agencies, especially in the case of more fragile settings where independent purchasing structures have been created, although in countries such as Zimbabwe, with stronger health systems, a degree of national accountability has been created. Limited improvements have been made to community engagement. However, the clearest changes to purchasing arrangements relate to providers, at least in relation to the sub-package of services on which RBF has focused.

**Table 3:** Summary of findings: impact of RBF on purchasing functions in Zimbabwe

*Key strategic purchasing actions by government*	Ensure adequate resources mobilised	•RBF provided modest but partially additional funds, still significant for primary care providers •Focused on MCH indicators •Donor dependent, though some Ministry of Finance copayments in later years
	Ensure accountability of purchaser(s)	•Parallel system with external purchasers •Accountability of purchasers to funders as well as to government •Bulk of purchasing continued through other channels, not affected
	Fill service delivery infrastructure gaps	•RBF provided some upfront investment, but no major revision of infrastructure planning in relation to needs
Key strategic purchasing actions *in relation to citizens/population served*	Inform the population of entitlements Establish mechanisms for complaints and feedback Publicly report on use of resources and performance	•RBF requires price list to be made public on the facility wall •Community satisfaction survey carried out as part of RBF verification •RBF helped revive Health Centre Committees and shifted their focus from resource mobilization to resource allocation; variable results and capacity
	Assess needs, preferences, values of the population to specify benefits	•No consultations on needs, values and preferences linked to RBF package •Package defined nationally with no scope for variation at local level
Key strategic purchasing actions *in relation to providers*	Establish service agreements/select (accredit) providers	•RBF did not change accreditation system •RBF required facilities to meet minimum criteria, including developing an operational plan, having a bank account and a functioning HCC •RBF introduced contracts; contracts are limited to services and facilities covered by RBF
	Developing formularies and standard treatment guidelines	•RBF worked within existing guidelines; no change introduced here
	Design, implement, modify provider payment methods to encourage efficiency and quality Establish provider payment rates Pay providers regularly	•RBF introduced payment rates for services (not the practice before in public purchasing) •Mixed picture in terms of outputs and quality improvements •Focus on MCH services, including some for which coverage is high, raising questions about efficiency; no local ability to adapt indicators •RBF functioning more as financing mechanism than incentive (partly because of wider budget cuts) •Payments driven mainly by catchment population size •Concerns over regularity and sustainability of payments (rates have been reduced over time); costs of RBF implementation high but reducing •Evidence of learning/iteration but RBF system is complex to adjust •Some quality improvements (e.g., drugs availability) and convergence on national tools for quality improvement (though still some duplication across programmes)
	Allocate resources equitably and implement strategies to promote equitable access	•Remoteness bonus, but considered too small and failed to compensate facilities with small catchment areas •Equitable design in terms of service package covered •Poor urban areas excluded
	Establishing and monitoring user payment policies	•RBF aimed to remove user fees for the MCH services it covered. However, no difference in incidence of out of pocket payments between control/intervention areas found in impact evaluation •Financial and non-financial barriers continue to be significant
	Securing information on services provided, and developing, managing and using information systems	•RBF brought greater focus on data quality, though still many weaknesses •System for penalizing poor recording may be too strict (causing unfair loss of revenues by facilities) •RBF used HMIS data after having verified and corrected it •Providers have multiple data reporting requirements
	Monitoring provider results and acting on poor results	•Pre-existing well developed and integrated supervision system to which RBF provided funding •Variation in robustness of supervision, linked to wider issues of leadership and resources •Considerable technical support required from implementers’ field officers (and challenges in institutionalizing that)
	Auditing provider claims and protecting against fraud and corruption	•Complex verification/counter-verification procedures for RBF, and restrictions on procurement (from public finance rules) •Little evidence of false claims, able to move to risk based verification; challenge of shift to longer term (integrated) controls

This case study illustrates the complexity of assessing health system reforms, particularly in dynamic contexts. The question of where the baseline is drawn is of particular importance. For those working in Zimbabwe over the last decade, RBF is part of a story of reconstruction and strengthening of the health system and its performance. However, for those with a longer memory of how the system operated pre-crisis, there is often a different perspective – of putting resources into a system which had once been highly functional and had not lost its residual capacity and professionalism [13]. In this second narrative, many of the problems observed post-crisis were due to a combination of resource constraints and short term measures introduced by partners, to assist in an environment where trusting relationships with government were limited. In this respect, Zimbabwe exhibited some aspects of fragility – notably low resource mobilisation and the associated challenges of managing donor funds – but not others (for example, retaining institutional and professional capacity in the health sector). Before the crisis, there was a strong planning function, with good economists and planners. As noted by the World Bank, the Zimbabwe system had been efficient pre-crisis in producing health outcomes at relatively low cost, although that efficiency had declined during the crisis [19]*.* This makes assessment of the contribution of RBF more challenging: was it simply enabling a functional system to operate or was it embedding necessary systemic reforms?

Assessing changes to purchasing and the contribution of RBF to make it more strategic is also challenging in light of the wide-ranging nature of this concept under the framework adopted for analysis. At a broad level, the volume of funding coming from RBF (an estimated $2 per capita [46]) was not sufficient to expect radical changes to structures and systems. Some aspects were however clearly appreciated, such as the more structured approach to supervision. We note a greater separation of the purchasing function, a reinforcement of community engagement, more attention to quality of data and increased primary facility autonomy over resources. Most of the changes occurred at provider level. The impacts on facility governance in the impact assessment were not significant however [14]. High external dependence for resources and fragmented purchasing – especially common elements in fragile and conflict affected settings [47] - are important constraints for the government and for RBF. The administrative weight of the RBF programme has also been a challenge to agility, as noted in other settings where verification and transaction costs have been high [48].

Reflecting on [43], we find that Zimbabwe followed a particular trajectory, with geographic expansion occurring before institutionalisation, which reflects the history and political economy of the policy [13]. It seems clear that RBF benefited from the managerial capacity in Zimbabwe, which also enabled the MoHCC to take ownership of the programme and to ensure its adaptation to context [13]. However, RBF has continued to function as a vertical programme with an MCH focus and relatively little engagement from other programmes, and has not contributed to a reduced fragmentation in the sector. This reflection is also found in a paper on health worker motivation and RBF in Zimbabwe, which concludes that introducing RBF arrangements cannot alone overcome chronic systemic weaknesses, which require explicit organisational change management processes to be put in place across the system [49]. A survey of health care purchasing in five (RBF and non-RBF) districts in 2014 [50] also highlighted some important outstanding challenges, including unpredictable and inadequate funding for key purchasing functions; weak knowledge in districts of available resources and weak planning; cost escalation and external dependency, making it difficult to build internal systems for efficiency and effectiveness; and variation in the funding of different categories of services, with non-communicable diseases having no prepayment arrangements in place and some conditions with rising health burdens not being effectively purchased, leaving users exposed to charges or restricted access to the service, despite treatment guidelines for services being in place in all institutions and treatment procedures and patient data being recorded.

The Health Financing Strategy highlights the on-going inadequate capacity in the MoHCC for strategic purchasing [25], and highlights weak control mechanisms for compliance (use of funds for the intended purposes) as well as performance (value of money and achievement of results) as one of its chief challenges. The overall approach to purchasing remains to be clarified, including how RBF will relate to the planned national health insurance system [25] and development of an essential health care package. Other assessments have highlighted the need for a central unit in the MoHCC focusing on performance management of the overall system [22]. In a companion piece, we highlight some of the critical choices facing the institutionalisation of RBF in Zimbabwe today [13].

## Conclusions

While RBF has been presented by some as a potential catalyst to wider health system reforms and as a tool to increase strategic purchasing, the example of Zimbabwe – one of the few countries in Africa to be implementing RBF on a nationwide scale and widely seen as a successful example of RBF – suggests that we should have more realistic expectations, at least in the short term. RBF in this case brought focus to specific outputs – MCH services at primary level – but remained one purchasing mechanism amongst many, with so far limited traction over the main service delivery inputs and programmes. Its achievements included enabling some flexible resources to reach primary providers, funding supervision and emphasising the importance of reporting. Set against that were many constraints, including implementation costs at different levels of the system and broader challenges facing the whole sector after a decade of disinvestment.

Zimbabwe’s economic and political crisis provided an important entry point for RBF, as a programme which could bring much-needed resources, but Zimbabwe did not present a ‘blank slate’ for RBF to reform: it was a highly functional health system pre-crisis, which both enabled relatively swift scale up of RBF but also meant that the potential for significant restructuring of institutional relationships in relation to purchasing was more limited. With RBF, Zimbabwe has taken some important steps in the direction of strategic purchasing, but many gaps remain and the country still has to emerge from its chronic economic challenges and define its vision for the future of strategic purchasing for health, and RBF’s place within that.

## Supplementary information

**Additional file 1.**

## Data Availability

The datasets generated and/or analysed during the current study are not publicly available due the difficulty of anonymising the content and the lack of permission from key informants for on-sharing.

## Abbreviations

DHE: District Health Executive
DMO: District Medical Officer
EU: European Union
FCAS: Fragile and conflict-affected states
HCC: Health Centre Committee
HDF: Health Development Fund
HMIS: Health Management Information System
HTF: Health Transition Fund
KI: Key informant
MCH: Maternal and child health
MoHCC: Ministry of Health and Child Care
PMD: Provincial Medical Director
RBF: Results-based financing
RDC: Rural District Council
RHC: Rural health centre

## Footnotes

1 http://www.healthfinancingafrica.org/home/strategic-purchasing-expectations-upon-purchasers; http://www.healthfinancingafrica.org/home/strategic-purchasing-2-the-central-role-of-stewards

2 Detailed information about the design and the evolution of the RBF programme in Zimbabwe against the background of the economic crisis can be found in [13]

3 https://www.rbfhealth.org/resource/zimbabwes-urban-voucher-programme

4 https://www.healthsystemsglobal.org/webinars/41/Performance-based-financing-in-fragile-and-conflict-affected-settings-from-research-to-practice.html)
