# Supplementary material for: Results-based financing as a strategic purchasing intervention: some progress but much further to go in Zimbabwe?
Source: BMC Health Serv Res. 2020 Mar 6;20:180. doi: 10.1186/s12913-020-5037-6 (PMC7059677; doi:10.1186/s12913-020-5037-6)
Supplement: Supplementary file 1 — Additional file 1. [file 12913_2020_5037_MOESM1_ESM.docx]

**Topic guide**

A. Note details of participants before interview:

| 1. Interviewee ID |  |
| --- | --- |
| 1. Date of Interview |  |
| 1. Gender | Male □ Female □ |
| 1. Title of interviewee |  |
| 1. Institution / Organization / Department |  |
| 1. Central, or Region & District |  |

SECTION A

Ask some general questions about how long the person has been in post, the time period of his/her experience with RBF in Zimbabwe.

Work experience:

- 1. What is your post?
  2. How long have you been in this post?
  3. How have you been involved with RBF and for long?

SECTION B.

Questions *(tailor according to background of KI and the period to which their knowledge and experience is relevant)*

1. **Framing of the concept**

What does the term “performance based financing” mean to you?

1. What do you think are the main changes that introducing PBF entails?
2. Which elements/domains of the health systems does it affect, how? (probe: e.g. HRH, financing of facilities, purchasing...?)
3. Has your understanding of the term changed over time (if so, how? why?).
4. **Period 2010-2011: adoption and design of RBF pilot phase**
5. When did the discussions about RBF start in Zimbabwe?
6. Who introduced them? (external vs. internal actors, etc.)
7. In which context? During which discussions, fora, etc.?
8. Why was RBF considered a potentially useful option and/or why was it opposed?
9. Who were the key actors involved in RBF during its adoption and design?
10. How were they positioned (in favour, against, neutral in relation to RBF)?
11. What was their relative power and influence and how was it exercised? What were their interests?
12. What other drivers and barriers could be identified for the adoption of RBF (other than actors – for example, features in the global or national context, challenges to be address in the health system, elements of ‘trust’, ‘decentralization’, existing policies and practices, ideologies and values held etc.)?
13. How were the design choices made concerning the RBF programme?
14. What features were most debated in relation to design? (e.g., choices concerning: national coverage, focus on health facilities, internal verification arrangements, etc.)
15. Did it build on or was it affected by any historical legacies in Zimbabwe? How?
16. Who proposed/opposed them (in general and in relation to specific arrangements)?
17. How did it affect overall sector resourcing, in your assessment?
18. **Period 2011-2014: implementation of RBF pilot**
19. How was the RBF pilot implemented?
20. What is your view concerning its strengths and successes?
21. What were the main challenges or bottlenecks to implementation?
22. What was the role in RBF implementation (and their views of RBF, and benefits or losses from it) of (*as relevant*)?

- Central level MoH’s department (*differentiate between departments if possible*)
- Other governmental bodies
- Donors and NGOs
- Provincial health executives
- District health executives
- Facilities (health centres and hospitals)
- The private sector
- Civil society

1. **Period 2014-16: scale up of RBF**
2. Why was RBF scaled up in 2014?
3. Which were the main actors involved in this decision? Any strong champions or opponents?
4. Who was empowered to take decisions?
5. How much political priority did the programme have?
6. Was there any opposition to the scale up?
7. What were the drivers and barriers (other than actors) during the scale up?
8. How was evidence used in decisions on the programme’s evolution? Where any trade-offs identified and if so, how were they handled?
9. What was the role of external actors? How well were they coordinated? What were their interests?
10. How was RBF altered at this stage (if it was) and why? Did roles change?
11. How would you assess the current nationwide implementation of RBF? Distinguish between schemes if relevant:
    - Key strength and success
    - Main challenges
    - Impact on costs and effectiveness of services
    - Impacts on the wider health system
    - Impact on equity and access for marginalized groups
    - Any winners or losers in terms of different institutions and actors
    - Impact on rent seeking (and if so, at what levels)?
12. **Post-2016: institutionalization of the RBF programme**
13. What is the current state of play of the RBF programme?
14. How do you think it will evolve in future?
15. What are your recommendations in relation to it, and why?
    - Its potential and limitations in terms of core health system challenges in Zimbabwe
    - How it might be adapted and sustained, as relevant
16. **Purchasing functions and RBF**

## Key strategic purchasing actions by government

Which bodies were undertaking health purchasing functions prior to RBF (MoH, other sectors, municipalities, other insurers etc.)? How clear were their roles? How have these changed as a result of RBF (and why/why not)?

This includes:

- assessing service delivery infrastructure gaps?
- mobilising resources to meet service entitlements?
- holding purchasers accountable?

## Key strategic purchasing actions in relation to population served

Who was assessing the service needs, preferences and values of the population and using them to specify service entitlements prior to RBF? How effective was this? How has this changed as a result of RBF (and why/why not)?

This includes:

- informing the population of their entitlements and obligations
- ensuring the population can access their entitlements
- establishing effective mechanisms to receive and respond to complaints and feedback from the population
- publically reporting on resource use and other measures of results

## Key strategic purchasing actions in relation to providers

Who used to select or accredit providers, considering the range and quality of services, and their locations? How effective was this? How has this changed as a result of RBF (and why/why not)?

This includes:

- Establishing service agreements/contracts
- Developing formularies (of generic drugs, surgical supplies, prostheses etc.) and standard treatment guidelines
- Designing, implementing and modifying provider payment methods to encourage efficiency and service quality
- Establishing provider payment rates
- Securing information on services provided
- Monitoring provider results and acting on poor results
- Auditing provider claims
- Protecting against fraud and corruption
- Paying providers regularly
- Allocating resources equitably across areas
- Implementing other strategies to promote equitable access to services
- Establishing and monitoring user payment policies
- Developing, managing and using information systems

1. What are the key strengths and the main challenges of the current arrangements in terms of purchasing functions?
2. Are there differences in purchasing across schemes or provinces, and why?
3. Are the current RBF purchasing arrangements sustainable over time, both financially and technically/institutionally?
4. How well integrated is the RBF purchasing within the other elements of the health system?
5. What impact has it had on wider health system purchasing arrangements?
6. Do you have any recommendations in relation to how RBF could strengthen overall strategic purchaser in the health system in Zimbabwe?
